# The Therapeutic Effects and Uses of Mercury, Etc.

**Published:** 1873

**Authors:** Wm. H. Doughty

**Affiliations:** Augusta, Ga., Professor of Materia Medica and Therapeutics in the Medical College of Georgia


### THE THERAPEUTIC EFFECTS AND USES OF MERCURY,

### *AS INFLUENCED BY THE "REPORT OF THE EDINBURG COMMITTEE ON THE ACTION OF MERCURY, PODOPHYLLIN, AND TARAXACUM, ON THE BILIARY SECRETION."*

BY WM. H. DOUGHTY, M.D., AUGUSTA, GA.,

*Professor of Materia Medica and Therapeutics in the Medical College of Georgia.*

[From the Transactions of the Medical Association of Georgia, 1873.]

(*Continued from August Number.*)

PROPOSITION III.—*That the Committee fly the issue which they have precipitated when, in reply to this, they assume to require "Objectors" to "prove that, in any case whatever, mercury can increase the biliary secretion in man."*

Having laid siege to what is conceived to be an impregnable position, namely, that mercury possesses cholagogue properties, like "sappers and miners," they proceed to undermine it by physiological experiments, when reaching the point at which the "mine" was to be sprung that would overturn and demolish the therapeutical uses of mercury in hepatic diseases, they suddenly discover that it is, after all, a mere "hypothesis," demanding even proof of anything real. This is decidedly "Quixotic," and shows how well they had regarded the advice of Sydenham to Sir R. Blackmore, to begin the study of medicine by reading Don Quixote.

How very unlike the course of Horatio C. Wood, Jr., who, conducting his experiments upon the "Physiological Action of Viridia, Veratroidia," etc.,* to the point where the results must be turned to therapeutical account, boldly avows that "*no experiments on animals are absolutely satisfactory unless confirmed upon*

*American Journal of Medical Sciences. Vol. lix.

*man himself.*" The committee, confessing in the next sentence that their experiments are *impossible* of confirmation in man (well or sick), proceed, however, to pronounce that an "hypothesis" which is thought to be a fact established by the only method capable of demonstrating it. They assume to require of the clinical world proof that, "in any case whatever, mercury can increase the biliary secretion in man," as if proof furnished by its therapeutic use was not abundant, and had not already secured, riveted the conviction, the universality and fixedness of which had prompted their labors. Verily, this discloses the weak point in their line of advance. Led to the point at which they are required to utilize the physiological conclusion that mercury *diminished the flow of bile*, in answer to the rational suggestion, that where the *conditions* were different, as entailed by disease, the effect might be altered likewise, they seek to bolster themselves in a negative position. This we call "flying the issue," or rather "begging the question." The therapeutical proofs of the cholagogue effects of mercury are indelibly written in the cherished place which the agent still holds in the minds of practical men everywhere, and this against the fiercest opposition, nurtured and strengthened by popular prejudice, ever encountered by any medicine. They will still turn to it with unquestioned confidence, not unmingled with fear, however, in this class of disorders, hoping to realize its benefits to the exclusion of its injurious possibilities.

In commenting on this report, Dr. Thomas R. Fraser,* in a well written, practical defense of the therapeutical application of mercury, remarks as follows:

"In order to prove the existence of this action, it is sufficient that *the quantity of bile discharged into the intestines may be increased by mercury;* but the experiments of Scott and the Edinburg Committee were performed in such a manner that *an action on the secreting activity of liver-cells*—not on the flow of bile into the intestines—*could alone be examined.* Therefore, it can never be shown by these experiments that mercury does not act as a cholagogue."

Finding, then, that the evidence is unsatisfactory, we pass to

*American Journal of Medical Sciences. p. 250. Vol. lxii.

PROPOSITION IV.—*That the Hepatic Uses of Mercury are not dependent upon a simple increase of the bile. It is not so regarded by those who employ it.*

The reader can not fail to note the emphasis that is placed upon the *mere increase* of the biliary secretion by the committee. As this was the compass of their experiments, it is not at all surprising that the cholagogue effects of mercury should have been limited to the augmentation or diminution of the secretion. The word cholagogue (*χολη*, bile, and *αλω*, I expel), authorizes no such restrictive meaning, and we are quite sure that the diversified uses of the agent by therapeutists, in even opposite morbid conditions of the organ, are not better calculated to restrain it. If this limitation of its powers be correct, then the statement above quoted from Dr. Fraser, namely, that "it is sufficient to establish that the *quantity of bile discharged into the intestines may be increased by mercury*," acquires new force. This may be technically sufficient to contravene the results of such experiments, but we believe that the mere establishment of an increased flow of bile into the *intestines*, would not meet the expectation of many, perhaps, most, of those who employ it. They look behind and beyond this incidental result, which is dependent upon the accidental accumulation of bile in the gall-bladder and biliary ducts, to a more direct influence upon the liver itself. But we do not intend to be led into a discussion of the mode of action of mercury upon the liver. The clinical observer is not bound to explain the *modus operandi* of his remedies, however desirable that may be, but he is bound to employ them when their virtues are clearly established, without regard to his ability to explain them. The cure of disease is the *ultima thule* with him.

It is not to be denied, however, that, in so far as writers upon Materia Medica and practitioners, reasoning from the therapeutical to the probable *physiological* effects of mercury, have assumed an increased secretion of bile as part thereof, an error has been indulged. The sensible effect on a healthy animal is such a disturbance as produces a decrease in the quantity secreted by the liver. Let the therapeutist recant this much, making due acknowledgment to the members of the committee for the light bestowed by experiment upon this point.

As an indication of the views of the profession touching the action of mercury upon the liver, we quote as follows, from the *United States Dispensatory:*

"In functional derangement of the digestive organs, mercurials in minute doses often exert a salutary operation, subverting the morbid action, and that too by their slow alterative effect, without affecting the mouth. In these cases, no decided disturbance of the vital functions takes place, but the alvine discharges, if clay-colored, are generally restored to their natural hue, a certain proof that the remedy is *stimulating the liver* and *promoting the secretion of bile.* Indeed, there is no fact better established in medicine than the influence of the mercurial preparations over the hepatic system, and whether the liver be torpid and obstructed, as in jaundice, or pouring out a redundancy of bile, as in melœna, its judicious use seems equally efficacious in unloading the viscus, or restoring its secretion to a healthy state."

The following extract from our lectures on mercury as a cathartic, is to the same effect:

"When it is said that mercury stimulates the liver or promotes the secretion of bile, you will not understand it as implying an *absolute increase over health*, or a literal *excess* of that secretion, but simply a flow approaching, in uniformity of characters, the quantity and quality of health. Excess of bile which is either pent up in the gall-bladder, or escaping into the digestive cavity, constitutes functional disorder, producing sometimes grave symptoms.

"Over all the vegetable and mineral cathartics, it possesses the advantage of a definite action upon the liver, and glandular organs generally, so that its proper action is always characterized by bile (or what is presumed to be bile) in the evacuations; i. e., they acquire a specific, recognizable character, called bilious, differing from ordinary evacuations. What are called bilious stools, or biliary evacuations, vary very materially, in their sensible properties, through the intestines (from the rapidity of its discharge; or of bile vitiated inquality, as it comes from the liver and its reservoir; or again, of the discoloration produced in either, during its passage along the intestines; they vary in color, consistency, and quantity. Now, when it is said that calomel produces bilious passages, you will understand that any of these varieties may appear. They may be yellow, or of a healthy, bilious character; or have a red or brick-dust appearance, (resembling those produced by the tomato), from some change in the coloring-matter; or be green from the discharge of bile of a more vitiated character, or acquired, perhaps, by reaction with acids in the bowels; or be black or dark-brown from the action of sulphureted gases on undissolved portions of the mineral, or from the ejection of bile itself, originally black and insoluble. In consistency they may vary from liquid, frothy dejections to those having the consistency of mush. Vitiated bile is an acrid fluid, and when, from any cause, it is brought into the digestive cavity, it excites purgation—may also vomit upon accidental regurgitation into the stomach—but the acrid substance brings along with it copious discharges of serum and mucus. The appearance of mercurial actions, you observe, will then depend upon the condition of the bile already secreted and collected in the reservoir, and upon the freedom of the intestines from acids, gases, as well as the presence of undissolved portions of the mineral."*

There is room to fear that the idea of stimulation, as embraced in the phrase so often quoted and used, namely, that mercury

*Now that the digestive solvent for calomel has been discovered, we are no longer under the necessity of ascribing the discoloration of the fæces to the reaction of the undissolved mineral. Hence, the expected *green* calomel stools deemed characteristic, will take their true position as incident to the failure of the agent in whole, or part, to be observed. It may be proper to state that Tuson's experiments on the digestion of calomel prove incontestably that the *gastric juice* is a solvent for the heretofore mysteriously *in*-soluble but active agent. It may not be uninteresting to the reader to recite briefly the experiments on this important subject:

EXPERIMENT 1.—A mixture of pure calomel and distilled water, containing two per cent of hydrochloric acid was placed in one vessel.

EXPERIMENT 2.—In another vessel a mixture of calomel, pepsine (Bullock & Reynolds) and distilled water.

EXPERIMENT 3.—In another vessel, a mixture of calomel, pepsine and distilled water, containing two per cent. of hydrochloric acid.

These mixtures were kept at 38° C. (100.2° F.) twenty-four hours, and occasionally stirred or shaken. They were then filtered, and the filtrates saturated with sulphuretted hydrogen. The filtrates from first and second remained unaltered, but that from the third gave a black precipitate of Hg. S.—*Richmond & Louisville Medical Journal, January,* 1873. Copied from *Medical Times & Gazette, May* 4, 1872, *and Pharmaceutical Journal, December* 23, 1871.

"*stimulates the biliary secretion,*" has misled some; hence, the looked-for increase or excess of secretion in physiological experiments. That mercury stimulates in the sense that alcohol excites, or that an irritant diuretic does (which may even light up nephritis), is a misinterpretation of the word; perhaps the use of the word "stimulate" is unfortunate in this connection. Headland, in his work on the "Action of Medicines," page 322, fourth American edition, is guilty of this error, when he says that a "large dose of mercury, naturally a cholagogue, may produce jaundice, by causing congestion of the liver." A melancholy illustration of the complete subjection of the mind of the author to a *theory* of the action of medicines upon particular organs, which he was seeking to establish.

The extract from the *United States Dispensatory* gives its true signification,—as a promoter, prompter, restorer, corrective of morbid qualities, etc. It matters very little, then, so far as its therapeutical uses are concerned, whether, in its physiological effects, it *increases* or *decreases* the flow. Here we would be content to rest the question, but for the fact that the committee, constructively at least, deny the possibility of making observations upon the influence of mercury on the liver. This brings us to the discussion of

PROPOSITION V.—*How Observations on the Effects of Mercury upon the liver may be made.*

The belief in the cholagogue effect of mercury is the result of clinical observation. The latter may, therefore, be taken as a test (or means of proof) of the action of the agent upon this organ separate and apart from the therapeutic end achieved by its use, provided the conditions and circumstances attendant upon its employment are of a character to give reasonable certainty and *direction* to it in said uses. Clinical medicine in this light has a two-fold aspect: It affords at once experiments upon the cure of disease, and the *modus operandi*, as well as effects, of remedial agents. The two questions or objects, although interlaced, are nevertheless capable of separate pursuit by observers in search of either. This mode of observation, liable as it is to misinterpretation, may be justly charged with being indirect, and, on this account, conclusions reached are necessarily uncertain. But let us not be guilty of the error of esteeming it too lightly because of its indirectness, particularly where surer methods are not available or applicable. Can no positive conclusions be safely arrived at by indirect modes of observation? How often does the physician employ the process of reasoning by *exclusion* in order to make a *certain* diagnosis—an end not to be achieved by other means or modes? Diagnoses thus made are conclusive, and acted upon without reserve by him.

But clinical observation, as a mode of experimentation upon the influence of mercury and other articles on the liver, loses much of its uncertainty when taken in connection with physiological and pathological data, themselves well calculated to influence correct conclusions. Without the confirmatory relations that these bear to the question in hand, it would be impossible to do more than conjecture the degree of influence of mercury upon the biliary secretion; with them, however, we are furnished with a guide that secures a close approximation to exactness and literal truth. To give point to the discussion, we would ask: *How do physiology, pathology, and therapeutical observation, combine to establish the cholagogue effects of mercury, so as to make them a full equivalent for direct experimentation?*

### PHYSIOLOGICAL CONSIDERATIONS.

In the first place, let it be remembered that the liver is the only organ, having relations with the alimentary tract, that furnishes a secretion having a tangible, distinctive pigment, not simply visible, but capable of chemical identification. Moreover, this secretion, more easily recognized by reason of its abundance, (calculated at about thirty-two ounces in twenty-four hours), while it is chiefly employed in intestinal digestion, its recrementitious portions being taken back into the circulation, yet furnishes excrementitious elements, the presence of which may be disclosed in the fæcal contents of the large intestines, giving color and character to them. The elements referred to are stercorine, a chrystalizable substance into which cholesterine is transmuted during the transit along the intestines, and *biliverdin*, which, when modified, becomes the coloring matter of the fæces. The fæces afford opportunity for estimating the presumably healthy action of all the digestive organs—the liver in particular—proper allowance being made for alterations of color due to particular articles of food or foreign materials gaining admission into the stomach.

But some one, objecting, will say that the biliverdin is not recognizable in the fæces, even by chemical tests, and possibly deny, therefore, that their color can be considered as throwing light upon the liver in its action. True, the biliverdin is altered in the small intestine during digestion, but not without leaving a modified pigment, derivable from no other known source, and therefore of equal value as an index of the earlier secretion of the liver. It scarcely admits of question, that the biliverdin itself, in disease where failure of digestion is so often seen, may become apparent in the fæces, having been hurried through, or escaping change in, the small intestines."*

But a few citations of facts from authorities may serve to clear up all doubt on this point.

Flint† traces the coloring properties of the bile upon food undergoing digestion, even to the fæcal remnant, in the following remarks:

"As the contents of the stomach are passed little by little into the duodenum, the chymous mass becomes of a bright-yellow color, and its fluidity is increased from the admixture of bile and pancreatic fluid. In passing along the canal, the consistence of the mass gradually diminishes, from the absorption of its liquid portions, and the *color becomes darker;* and by the time that the contents of the ileum are ready to pass into the cœcum, the greatest part of those substances which we recog-

*"Although Simon found that the normal fæces contained little, if any, biliary matter; he discovered in them 21.4 per cent. of the organic principles of bile after the operation of a large dose of calomel."—*Headland on Action of Medicine, p.* 357, 3*d Edition.*

Niemeyer, in his "Text-book of Practical Medicine," page 547, in treating of "intestinal catarrh," says: "At first the evacuations are more mucous, and less copious, than natural, have an acid reaction, and immediately after being passed, or after being exposed to the air for a while, they have a greenish color. This depends on the admixture of unchanged bile, and on higher oxidation of the still retained coloring matter of the bile."

On page 544, he remarks: "The color of fluid stools is usually some shade of green; this does not depend on an abnormal quantity of bile being poured into the intestines, but on the bile being evacuated with the fluid and the intestinal secretions, before it has undergone the normal changes,"

†See Flint's Physiology—Article, Digestion—page 391.

nized as alimentary principles have become changed and absorbed.

"Passing the ilio-cœcal vale, the color changes from the yellow, more or less bright, which is observed in the ileum, to the dark, yellowish-brown characteristic of the fæces. Though the bile pigment can not usually be recognized by the ordinary tests, it is this which *gives to the contents of the large intestines their peculiar color, which is lost when the bile is not discharged into the duodenum.*"—(Page 394. Italics are ours.)

Again, in his observations on a dog with a biliary fistula, he says that after the second day the alvine discharges "became very infrequent, though the animal ate well every day. The fæces that were passed after the third day were of a grayish color, and moderately soft. They had an exceedingly offensive and penetrating odor. At about the fifteenth day the fæces became more frequent, and from that time were passed three or four times a day. Generally they were *clay-colored*, but on one or two occasions were quite dark. They always had a peculiarly offensive odor."—(Page 369.)

Again, Professor Busch of Bonne, in his physiological experiments on the digestive action of the intestinal juice, in a case of fistulous opening into the smaller intestines* at their upper third, the result of an injury, after speaking of the "*grass-green aspect*" of the chymous material which exuded from the upper end of the severed gut, says:

"The digestive property of the intestinal juice has been denied by some, while others have correctly admitted it. At the commencement of the treatment, the state of the patient began to be improved only when she was fed through the lower opening of the intestine. Into this were introduced soup, beer, gruel, hard eggs, and meat cut into small pieces. During the six weeks previous to her entrance into the hospital, the woman had only two small, hard stools. After being fed in the way above described, she had, at first, an abundant evacuation every twenty-four hours; but later, it became necessary to use *enemata*. The evacuations resembled ordinary fæcal matter in *form and consistence*, but the *color was grayish-white*, and they

*See July number American Journal of Medical Sciences, 1860. p. 217.

emitted a repulsive odor. No portions of egg or meat were found in them."—Page 218, July No., 1860.)

While this case illustrates the digestive power of the intestinal juice most forcibly, as well as that bile is not the *purgative* of the intestinal secretions, it also substantiates the fact that the biliary coloring matter is essential to the production of the ordinary healthful appearance of the fæces.

We direct attention to another case of intestinal fistula—an artificial anus—located at or about the junction of the cœcum and colon, reported by Dr. William Hunt, in Pennsylvania Hospital Reports for 1868 (page 175). Such was the size and location of the opening, that almost the entire contents of the small intestines escaped here, so little passing by and into the colon and rectum as to produce "a discharge from the rectum at *intervals of three or four months.*" He, too, made physiological observations and experiments. Let us see of how much value in the present inquiry:

"The amount of fæcal discharge was at least from five to eight ounces daily, and certainly equaled that discharged by the rectum in a normal condition. Food of a laxative property would sometimes produce diarrhœa. The form of the fæces was mostly that of a soft mass, but sometimes there would be lumps of well-formed matter. An important point is here settled as to the functon of the colon,—it must be but a sacciform receptacle for the excrement, and possesses little or no secretive power of its own.

"*As to color and odor, there were no differences to note from normal fœcal discharges by the rectum.* They differed according to the food and state of the health of the patient, precisely as in other persons. As the opening was at the beginning of the colon, the passive character of this viscus is still further proved by this observation."—(Page 168).

We might multiply similar physiological observations, but none could more forcibly demomstrate the fact which we seek to avail of, namely, that the color of the fæces, dependent as it is upon the secretion of the liver, reflects, to a valuable degree, the freedom of the flow of bile into the intestines, enabling us thereby to arrive at just conclusions as to the state of the *liver*

*itself.* Other characters might be taken into the count, but this is the most salient, and least questionable.

### PATHOLOGICAL CONSIDERATIONS.

When disease invades the abdominal viscera, the uniformity of the condition that obtains in physiology is distributed, but with some limitation, the same princple of interpretation applies. Hence the value that attaches to a careful inspection of the fæcal dejections by physicians generally. It means something more than idle curiosity, or purposeless sight-seeing—it is the peering of the eye of science into the working of those inner associated organs, whose regularity of action and perfect working are judged of by the matters defecated. The large and small intestines and liver, respectively, impress characters upon them which physicians read at a glance, and with the manner of evacuation, etc., is even aided in his diagnoses. It is not our purpose, however, to discuss particular morbid conditions of any of these organs,—it is enough for us to show that alterations in the quantity and quality of the bile occur in the biliary passages and intestinal contents, similar to those that mark the altered character of the fæcal evacuations, and thus justify, presumptively, conclusions as to the proper, or improper actions of the liver itself. If we admit this, it may at once be claimed, that in so far as these morbid characters are produced, changed, or removed by particular medicines prescribed for the purpose, we are influencing the liver itself, thereby establishing a *cholagogue* effect. We may illustrate it in this way: Given a certain character of the evacuation with light furnished by the physiology of digestion, how can the healthy characters be restored, except by some agent capable of influencing the liver itself? The fact that it does so, establishes its cholagogue property. If bile is absent, or very deficient from the fæces, as is characteristic of jaundice, and mercury restores it, perhaps, after the administration of other remedies in vain, how are we to avoid ascribing to the agent a cholagogue property, no matter how exerted—whether directly or indirectly, i. e., by duodenal impressions, or direct contact with the liver-cells? Or if the biliary passages be filled with morbid bile, which, under the employment of mercury is more freely discharged, and healthy bile substituted therefor, it is not proper to assert the power of the agent to influence the organ at fault? How can we err in doing so?

Morbid bile is unfit for digestive purposes. Indeed, disturbs digestion. It is swept along with undigested matters to form a part of those morbid characters observed as well in the ileum as in the colon. Although liable to change of color from reactions had in its transit through the intestines, it is not always so, being sometimes seen in the latter (if the latter also after defecation) unchanged from the characters originally possessed when it flowed into the intestines. The same may be said, also, of healthy bile, which, in certain diseased states of the intestinal canal, is hurried so rapidly through it as to appear in the dejections as bile of good quality.

That the variations in the character of bilious evacuations may be due rather to morbid qualities of the bile than the reactions of the mercurial with the bile itself, or other intestinal secretions, we submit the following extracts from writers on pathological anatomy, going to establish the varied morbid qualities seen as well before as after death.

Rindfleisch,* speaking of the deviation of the bile pigment from the haematoidin of the blood, uses the following language:

"The senescent red blood-corpuscles lose their coloring matter, which imparts itself to the serum, from thence it is taken up into the liver-cells, converted into biliary-coloring matter, and removed from the body with the excrement. Previous to this removal, however, it undergoes, especially by long retention in the gall bladder, a further metamorphosis into yellow, green, brown and black shades, which Stadeler designates as bilifuscin, (C32 H20 N2 Os), biliverdin (C32 H20 N2 O10), biliprasin C32 H22 N2 O12), and bilihumine. We observe that bilifuscin distinguishes itself from bilirubine by containing two atoms more of H O, the biliverdin from bilifuscin by two more atoms of O, biliprasin from biliverdin, again by two atoms of water, while bilihumin is a black, insoluble, very highly oxidized body."

Is not the bile of these characters—particularly the latter, "black, insoluble, very highly oxydized"—discharged by vomiting and purging sometimes? Both spontaneously and after the administration of medicinal agents.

*Pathological Histology, p. 62.

Again, Rokitansky,* under the head of "Abnormities of the Biliary Passages," remarks:

"The bile itself presents great varieties as to quantity, but more still as to quality; in the majority of instances, the anomaly has its origin not so much in disease of the liver as in morbid conditions of other organs, especially of the intestine and portal blood.

"As regards quantity, the bile is found accumulated to a large amount in the biliary passages and intestine, or it is remarkably scanty. It is to be observed that in the latter case, the deficiency is sometimes compensated by the saturated condition of the fluid.

"The qualitative anomalies of the bile are more numerous and important, and affect both its physical and chemical constitution.

"The color of the bile varies extremely. It may be pale yellow, ochrey, orange-colored, yellowish brown, blackish brown, black, or all the different shades and tints of green. The consistency of the bile generally increases in a ratio with the increased depth of color, varying from the fluidity of water to the density of tar and of calculous concretions. In taste, it varies as to the amount of bitterness, but it may also be more or less, or entirely, sacharine, saline, sour, alkaline, acrid or insipid."

On page 93, he says:

"The color of the fæces mainly depends upon the color and degree of saturation of the bile. They may be dark brown, dark green, black, pitchy, or, in the absence of bile, grayish or clayey."

These considerations confirm the application of the principle that the *intestinal dejections*, which, according to Dr. William Hunt, are about the same before as after defection, *constitute a good criterion of the action, morbid or otherwise, of the liver.* With or without the use of medicines (except when known reactions with the latter would disguise it), pathological considerations contribute something toward determining the experimental effects of remedies upon the liver, by the altered character of alvine dejections due to admixture of bile.

*Path. Anatomy, vol. ii. p. 128-129.

Therapeutical observation assigns to mercury the property of conferring biliousness upon the evacuations: when bile is deficient or absent, the ordinary conditions existing, it sooner or later restores it; when superabundant, or vitiated in quality, it provides a healthier flow in quantity and quality. Confirmed as this is by the foregoing considerations, clinical observation affords almost direct experiments upon the liver itself. A rational therapeutical proceeding is thus firmly established upon the broad basis of physiological, pathological and clinical observation, and if mercury was the only eliminative capable of exerting wholesome influences upon the liver and its secretion, in their rightful relations to pathological conditions, we would be compelled by every consideration of justice and humanity to give it precedence over all others. With those who do thus exalt it, we shall not quarrel. They are entitled to the selection, provided they invest its use with safety and caution. In its indiscriminate habitual employment, it is like a two-edged sword, cutting asunder the vitals of the patient, at the same time that it corrects temporary derangement. It is a fearful agent in the hands of the multitude. During the late war, the Surgeon General of the United States army struck it from the supply table, not that he doubted its power for good, properly directed, but he thought it would be humane to sacrifice the few who would be benefited by its use, for the many who would be injured, to the serious detriment of the public service. We do not employ it except on rare occasions, because we have found other and safer remedies—not that we deny its cholagogue properties. We have defended it against unsatisfactory experiments, for the sake of truth. If, however, what we have written should serve to further its indiscriminate use, by those who give undue prominence to it as an *essential* agent in malarial fevers, hepatic disorders, indigestions, etc., let the blame rest upon those who thus abuse it. The way to restrict its therapeutical employment within the limits properly assigned it, is to disseminate sounder views of pathology, and a better understanding of the mode of action and application of other eliminatives and alteratives, rather than by abuse of those who give it, or by a denial of its known cholagogue properties.

The Edinburg Committee would impeach the views above presented, as "unfounded assumptions and vague speculations,"

declaring that their experiments alone furnish "unquestionable data." With due deference to them, however, we venture to assert that, until other evidence than that offered by physiological experiments is presented, mercury will still remain a valuable agent in hepatic disorders—one capable of much good, when properly used, but equally potential for mischief when abused. The conclusion to which we are brought, therefore, is that the therapeutic effects and uses of mercury remain, as heretofore, uninfluenced by the experimental evidence adduced by said committee.

---

Death of the Distinguished Surgeon, Nelaton.—The death of Dr. Auguste Nelaton is announced in the cable dispatches. From a daily newspaper the following notice is clipped:

"Dr. Auguste Nelaton, one of the most famous physicians of France, whose death has been announced in our cable dispatches, had completed his sixty-sixth year, having been born on the 17th of June, 1807. He was a pupil of Dupuytren, received his medical degree in December, 1836, and soon after was surgeon to the hospitals and a member of the Faculty of Medicine. In 1851, he was appointed Professor of Clinical Surgery in the University, and in 1856 was admitted to the Academy of Medicine in the section of Chirurgical Pathology. Originally decorated with the ribbon of the Legion of Honor in 1848, he passed through its various grades to the position of commander. He was chosen a member of the French Academy of Sciences in 1867, and soon after relinguished his professorship on account of feeble health. He was the author of several works on medicine and surgery."

